# Physical Activity Surveillance in Adolescents with Type 1 Diabetes: A Pilot Mixed-Methods Investigation

**DOI:** 10.1155/2022/4202561

**Published:** 2022-03-15

**Authors:** Susan Giblin, Paul Scully, Julie Evers, Niall Dalton, Grainne Hayes, Alan Donnelly, O. Orla Neylon, Clodagh O'Gorman

**Affiliations:** ^1^Department of Paediatrics, School of Medicine, University of Limerick, Ireland; ^2^Department of Paediatrics, University Hospital Limerick, Ireland; ^3^Department of Physical Education and Sports Science, Physical Activity for Health Research Cluster & Health Research Institute, University of Limerick, Ireland

## Abstract

Type 1 diabetes (T1D) affects over 2,500 children in Ireland. Insulin replacement is the mainstay of treatment for T1D, and physical activity (PA) is an important, modifiable lifestyle factor for sustaining health. Surveillance of PA for both research and clinical purposes in paediatric T1D has been limited. This study deployed both quantitative (accelerometry) and qualitative (self-report) measures to assess habitual PA patterns in children with T1D. Twenty-one participants (9 females, 12 males) between 10 and 17 years (mean 13.7 ± 1.94 years) were recruited from an Outpatients Paediatric Diabetes Clinic. Total steps, standing time (minutes (mins)) and sitting time (mins) were recorded using the activPAL 3 microactivity monitor. Clinical parameters (HbA1c, insulin regimen, and weight centiles) were measured. A self-report diary was used to measure perceived activity levels. The findings of this study show that participant children with T1D are not achieving the required steps per day to sustain physical health (recommended minimum 11,500). Females (mean = 7,306 steps ± 5,468) achieved significantly less (*p* = 0.001) steps per day compared to males (10,806 steps ± 5,904). No significant differences were found between genders for sitting time or standing time. Overweight or obesity was identified in 44% of female participants and 15% of male participants. Mean HbA1c for both females 8.25% (67 mmol/mol) and males 7.97% (64 mmol/mol) was above the International Society for Pediatric and Adolescent Diabetes (ISPAD) recommended <7.0% (53 mmol/mol) for children. Further research is warranted to investigate PA promotion strategies in populations of children with paediatric T1D.

## 1. Introduction

The purpose of this research was to pilot methodologies for further empirical research examining PA behaviours in children and adolescents with type 1 diabetes (T1D). T1D is a common, chronic, life-long illness with multifaceted considerations for the physical, psychological, and social implications associated with living with the condition [1]. Whilst insulin is the cornerstone of management for T1D [2], additional nonpharmacological interventions promote positive clinical, psychological, and social outcomes for chronic disease management [3]. Specifically, physical activity (PA) has been identified as an important adjunct to pharmacological management for T1D [2–7].

For healthy populations, there is a substantial amount of scientific evidence promoting the physical and psychological health benefits associated with living a physically active life-style [3–7]. Increased PA levels have been shown to improve cardiovascular fitness, bone health, and blood lipid profiles; reduce blood pressure; increase insulin sensitivity; and reduce the risk of comorbidities associated with sedentary lifestyles [3–7]. Consequently, the World Health Organization has issued a global plan on PA (2018–2030) [3] that calls on member states to increase mass participation in PA and reduce sedentary behaviour. PA engagement during childhood appears to play a mediating role in PA engagement and subsequent attainment of health-associated benefits later in life [4]. Thus, early intervention to promote PA engagement seems logical and even imperative in childhood. Unfortunately, people with chronic diseases tend to have fewer opportunities to access safe and appropriate PA programmes [3].

Despite the potential benefits of PA engagement [2–7], figures suggest that, similar to their healthy counterparts, children with T1D are not meeting the recommended 60 minutes of moderate-to-vigorous PA (MVPA) per day [7]. PA engagement for children with T1D requires careful management of blood glucose excursions [8]. Thus, T1D populations face significant, disease-specific barriers to PA engagement, and guidelines for safe participation in PA are warranted [8–12]. To date, research examining PA behaviours in paediatric T1D populations has focused on either qualitative investigations of self-reported PA levels and/or laboratory-based cross-sectional intervention studies [8]. Both approaches offer important preliminary insights, but there is a notable lack of empirical, ecological evidence available to bench-mark PA levels in T1D populations. Studies to date have varied in both methodology and duration; thus, comparative analysis between study outcomes is limited. Without comparative analysis, formulation of evidence-based strategies for PA promotion in T1D is inhibited.

Presently, technology is available and accessible to gather device-based information regarding PA levels in children with T1D [13–16]. For example, the accelerometer has been used to gather objective, large-scale, and longitudinal information on activity behaviours of the general population. The quantification of PA is used widely in the promotion of PA guidelines (e.g., recommended daily steps or activity minutes) [8–12]. Thus, data derived from device-based measures of habitual activity behaviour is required to examine the interrelationship between activity, sedentarism, and clinical parameters, e.g., HbA1c and anthropometrics in T1D populations. Without empirical habitual PA data, there are substantial gaps in our knowledge regarding PA patterns in paediatric T1D populations. For example, in the general population, gender differences in PA exist with a notable decline in female PA engagement across the lifespan [17]. The identification of patterns in T1D PA engagement profiles could aid in the targeted promotion of PA for at-risk subpopulations during crucial periods of development, for example, adolescence [17].

ISPAD guidelines (2018) [11] state that PA activity recommendations for children and adolescents with diabetes are the same as the general population. Fuirthermore, ISPAD provide clear recommendations to support children with diabetes in being physically active (e.g., timing of exercise, duration, intensity activity duration, and insulin dosing adjustment around exercise) [12]; however, it is unclear if PA recommendations are being achieved by paediatric populations with T1D. Thus, the purpose of this study was to pilot a mixed methods qualitative and quantitative protocol for assessing habitual PA data in children with T1D.

## 2. Methods

### 2.1. Participants

A cross-sectional observational study design was used to examine levels of PA in children with T1D.

Twenty-one adolescents (12 male, 9 female) were recruited from a paediatric diabetes outpatients' clinic in the midwest region of Ireland. Participants were between the ages of 10 and 17, were native English speakers, and were without comorbid injury. Only children with a diagnosis of T1D for greater than 12 months were eligible for inclusion. All participants and their parents were provided with parental and participant information sheets, consent forms, and a physical activity readiness questionnaire (PAR-Q). Participants were selected for inclusion once participant assent and parent consent were provided and they had successfully completed the PAR-Q. Ethics committee approval was granted by the Faculty of Education and Health Sciences Research Ethics Committee at the University of Limerick (REF 066/18).

### 2.2. Accelerometer Measurement of Habitual Activity Behaviour

**Figure 1:**
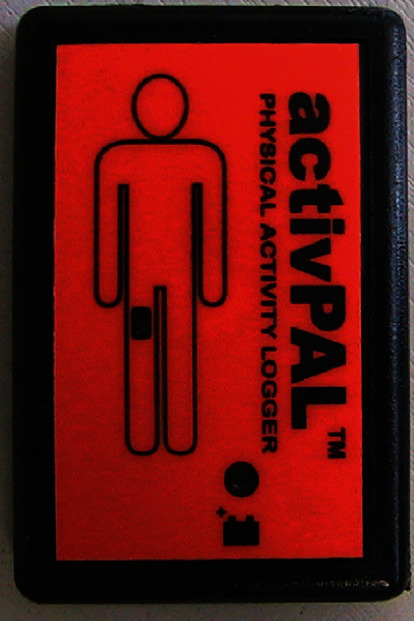
ActivPAL 3 microdevice.

Participants were provided with the activPAL 3 microdevice (AP3M) shown in Figure 1 and were encouraged to wear it 24 hours a day for eight consecutive days (PAL Technologies, Glasgow, Scotland). The device was waterproofed using a nitrile sleeve and affixed on the midpoint of the anterior aspect of the right thigh using a Tegaderm dressing. Participants were advised to remove the device *only* when engaging in prolonged water-based activities (i.e., swimming or bathing) or when playing full-contact sports (i.e., rugby). All monitor removals were documented in a nonwear diary. The output files from the AP3M were examined to calculate daily waking sedentary time, standing time, and stepping. Sedentary time and standing time were calculated using the postural function of the AP3M, using proprietary software (PAL Software Suite version 8). The number of steps was also taken directly from the activPAL output. To allow for accessible monitoring, the equivalent MVPA in daily steps has been previously researched [12]. Although there is some discrepancy between studies due to age categories, weight categories, and accelerometers/pedometers used, 11,500 steps per day have been identified as a target for both male and female children and adolescents [12, 18]. The mean time spent in each behaviour daily for each participant (weekdays and weekend days) was calculated. In accordance with prescribed accelerometer methodologies, for a day to be categorized as valid, participants were required to wear the activPAL device for a period of ≥10 hr during waking hours [13–16]. To be included in the final analysis, participants were required to provide at least four valid days of accelerometer recording (3 weekdays and 1 weekend day). All monitor outputs were examined for nonwear time, which was defined as a period with ≥60 minutes of consecutive zero accelerometer activity counts during waking hours. Participants completed a self-report activity diary for the duration of the wear protocol. Participants recorded daily wake time, sleep time and nonwear time. This information was cross-referenced with the self-reported nonwear time. The activPAL nonwear time was summated for each day and the 24 hours adjusted accordingly (24 hour-nonwear hours) [13–16].

### 2.3. Self-Report Measure of Habitual Activity Behaviour

Participants were asked to self-report their activity levels during the 8-day data collection using a self-report diary. Participants were asked to quantify the duration of time they spend in MVPA each day using 7 categories (none, 0-30 mins, 30-60 mins, 1-2 hours, 2-3 hours, 3-4 hours, or >4 hours). Participants were instructed that MVPA referred to any activity that caused them to sweat, breathe hard, or made their legs tired. Participants were asked to include activities that they had participated in with friends, family, an organised team, group, or club. School-based physical education was not included as the study was conducted during the school summer holiday period.

### 2.4. Measurement of Clinical Parameters

Anthropometric indices were obtained by trained staff at recruitment using standardized protocols [10]. Weight and height were recorded to the nearest 0.1 kg and 0.1 cm, respectively, using standardized procedures [10]. Body mass index (BMI) was calculated and converted to BMI percentiles on the basis of age and sex in accordance with the Centres for Disease Control and Prevention reference data [10]. BMI percentiles were used to categorize healthy weight (5^th^–85^th^ centile), overweight (86^th^-90^th^ centile), and obesity (>95^th^ centile) in accordance with the Centres for Disease Control and Prevention recommentations [10]. Capillary blood HbA1c was taken by a finger-prick blood sample and was analysed on a DCA Vantage Analyzer (Siemens/Bayer, Germany) in the clinic at the time of outpatient visit.

### 2.5. Statistical Analysis

SPSS version 25 for Mac was used for further analysis. Descriptive statistics, box plots, and histograms were used to examine the characteristics and distribution of data. Shapiro-Wilk's test was used to examine data distribution, and Levene's test was used to examine equality of variances. Independent samples of Student's *t*-tests were used to examine the differences between male and female step count, standing, and sitting minutes. Statistical significance was set as *p* < 0.05. Confidence intervals were set at 95%. Cohen's *d* was used to examine of effect size.

## 3. Results

Female and male participants' mean age, mean years since diagnosis, mean HbA1c level, and current pharmacotherapeutic management regimen (multiple daily insulin injections or insulin pump) are presented in Table 1. Multiple daily insulin injections were the most common pharmocotherapeutic regimen currently used by female (75%) and male (62%) participants. Mean HbA1c level for both female and male participants is above the ISPAD recommended level < 7% (53 mmol/mol).

Table 2 presents mean daily steps categorized by gender and weight classification (normal weight, overweight, or obese). A higher proportion of female participants (44%) were classified as overweight or obese compared to male participants (15%). For both genders, healthy weight participants recorded higher mean daily steps compared to overweight or obese participants, although differences in mean steps between weight classifications did not reach statistical significance (Table 2). The majority of male participants (66%) achieved the minimum recommended daily step count (11,500) on 4 or more days during the 8-day wear period compared to 33% of females (Table 3). On average, females achieved significantly less (*p* = 0.01) steps per day compared to males (mean = 7, 306 steps and 10,806 steps, respectively) (Table 4). No significant differences were found between genders for sitting time or standing time (Table 4). No significant differences were found between weekday and weekend step count, standing time, or sitting time (Table 5). All participants self-reported achieving a minimum of 30-60 minutes of MVPA (Table 6).

## 4. Discussion

The purpose of this study was to pilot a mixed-methods approach to investigating PA behaviours in children with T1D. The results show that on average, participants with T1D are not achieving the required steps per day to improve health parameters: Daily step count (8,220 ± 5,385) per day was on average below the recommended 11,500 steps per day estimated to equate to 60 minutes of MVPA for children and adolescents [12, 18]. Females (mean = 7,306 steps ±  5,468) achieved significantly less (*p* = 0.001) steps per day compared to males (10,806 steps ± 5,904). The findings are inkeeping with previous research reporting that children with T1D are not achieving the minimum recommended daily activity levels [5–7, 11, 18, 19] and provide quantitative information to supplement previous study findings that have relied solely on self-report measures. For example, one cross-sectional case control study [7] used self-report diary and questionnaire to compare activity profiles of children and adolescents with T1D (*n* = 138) with their healthy peers (*n* = 269). Similar to the findings in the present study, lower MVPA levels were reported in T1D participants and females reported lower MVPA levels than males [7]. Low PA levels in children with T1D may perpetuate low PA levels through adolescence and adulthood [14, 17].The Childhood Determinants of Adult Health (CDAH) study examined the effect of behavioural, attitudinal, sociocultural, and physical factors on PA behaviors in healthy children. This large population-based study with a 20-year follow-up showed that childhood and adolescent factors did influence PA behaviors during this transitional life stage. Perceived sports competency for females and physical fitness for males were found to be significant predictors of persisting with PA into adulthood [17]. Given the lower levels of PA reported in this study and elsewhere [5–7, 11, 18, 19], in T1D populations, interventions that target PA promotion and sedentary behaviour reduction in females with T1D during childhood years seem pertinent. A collaborative strategy where public health organisations, schools, and sports clubs work together to increase the skills, knowledge, and motivation required for sustained participation could help reduce the PA level reduction often seen, particularly in females during adolescent years.

Notably, in the present study 44% of the female participants and 15% of the male participants had BMI centiles in the overweight or very overweight categories. Average daily steps for both male and females were lower in participants classed as overweight and obese (male = 10,299 ± 5,544; female 4,742 ± 4,065) compared to healthy weight participants (male = 11, 742 ± 5,904; female = 7,756 ± 4,766), although differences between the weight categories for males and females did not reach significant levels (*p* = 0.91 and *p* = 0.34, respectively). These findings are in support of trends reported elsewhere [20, 21] where youths with T1D are more likely to be overweight or obese compared to their peers. Figures over the last 30 years show a twofold increase in the number of children with T1D who are overweight or obese [19]. Overweight and obesity present additional complications for youth with T1D such as higher risk of cardiovascular pathology and metabolic derangement [17]. Several case studies have reported children with T1D who are overweight or obese becoming resistant to their exogenous insulin and developing “double diabetes,” explained as a pattern of increasing insulin resistance on a background of insulin-requiring T1D. PA is a modifiable lifestyle factor that has been shown to manage weight in the general population; however, the relationship between PA and weight in individuals with T1D is less clear. PA promotion strategies that successfully translate PA policy into real-world PA engagement for children with T1D (via healthcare professionals, parents, and educations) could help reduce the risk of overweight and obesity in childhood and adolescents [8–12, 19].

Mean HbA1c levels for both females 8.25% (67 mmol/mol) and males 7.97% (64 mmol/mol) were above the ISPAD recommended <7.0% (53 mmol/mol) for children. The ISPAD expert consensus guidelines recommend the target of <7.0% (53 mmol/mol) to reduce adverse complications of chronically elevated blood glucose [20–23]. The Diabetes Control of Complications Trial (DCCT) and the Epidemiology of Diabetes Interventions and Complications (EDIC) report that chronically elevated HbA1c levels in children can increase a child's risk of experiencing adverse complications of diabetes such as retinopathy, neuropathy, renal impairment, and premature cardiovascular disease [22] . There is a large body of epidemiological evidence to suggest that MVPA is associated with more favourable glycaemic control profiles and less variation in HbA1c profiles [24]. Furthermore, there is evidence to suggest that the association between HbA1c levels and MVPA is present regardless of gender, age, pubertal status, body composition, insulin dose, and insulin regimen [24]. Higher levels of MVPA are required to achieve an improvement in aerobic capacity, and a higher aerobic capacity reduces the increase of insulin resistance risk factors in adolescents with type 1 diabetes [24]. Therfore, we hypothesize that increasing MVPA levels in children with T1D could reduce the risk of disease-associated complications later in life. For example, cardiovascular disease is the most frequent cause of premature death and disability in T1D [2], and children with T1D are at a higher risk of early adult-onset cardiovascular disease [2]. Previous studies have shown that adolescents with T1D have alterations in peripheral vascular function and myocardial parameters with early changes in blood pressure, peripheral vascular function, and left ventricular myocardial deformation indices [2, 3]. As PA can improve cardiovascular fitness, endothelial function, and vascular health in children living with T1D early, PA intervention aimed at increasing MVPA levels should be introduced to improve modifiable cardiovascular disease risk factors in this population [3, 24].

The findings of our study indicate a need for further education and intervention to promote appropriate activity engagement for this population group. For example, educating patients about the intensity and duration of PA required each day to achieve health benefits is warranted. Educational intervention including prescribed, individual exercise protocols to support patients in achieving the recommended daily requirements of MVPA could be beneficial. Additionally, wearable PA trackers could enable real-world monitoring of activity levels in children with T1D.

We suggest that our pilot investigation demonstrates that limitations of relying solely on self-report PA methods could be overcome with the further integration of device-based measures of PA for both the monitoring and promotion of PA daily recommendations [25]. Although commercially available PA trackers have gained popularity, integration of PA trackers into public health promotion policy has been limited, with the exception of Singapore's Health Promotion Board who introduced the first population-level wearable PA tracker initiative that encouraged increased daily step count and MVPA using reward and reinforcement for sustained behaviour change [25]. Meta-analysis of intervention studies using PA trackers for health promotion found that PA trackers are effective for improving daily step count and MVPA but do not impact light physical activity levels or sedentary behaviour levels [25]. Future research is advisable to explore the impact of wearable PA tracker interventions on MVPA levels and sedentary behaviours in T1D populations. In addition to providing empirical information to further understanding about the relationship between PA level and clinical diabetes parameters, wearable PA trackers effectively improve conscious exercise behaviour, including daily steps and weekly MVPA through the development of self-monitoring and self-regulatory behaviours [18, 25]. Activity monitoring could provide additional information that could be used in the outpatient clinic setting to provide individualised advice and recommendations about PA management.

## 5. Limitations and Future Directions

There are a number of factors that warrant consideration in interpreting the results of our study. For example, the weather was poor during the study period which may have limited outdoor activities compared to normal. Participants were monitored during their Summer holidays therefore the findings cannot be generalised to habitual activity patterns during school-term times. The self-report measure only quantified MVPA duration, future research to examine light PA, and sitting time is advised. Although there are acknowledged limitations of the study, the use objective assessment of activity is a strength that provides empirical information on activity patterns.

The primary strength of this study was the methods employed to accurately and objectively measure free-living activity behaviour and the collection of clinical parameters in a mixed child T1D population. Our results have implications for public health policy and physical activity counselling; however, the limitations of this study must be acknowledged. A noted limitation is the cross-sectional design, precluding causal inferences. Although every effort was made to randomly select participants, the study sample was small and the majority of the included participants were classified as adolescents. Further exploration in younger and older paediatric populations is warranted. While accelerometers are promoted for use in child PA research, they may not capture all activities that children partake in such as swimming, cycling, and load-bearing activity. Another limitation is participants' reactivity to wearing the device. While efforts were made to instruct participants not to alter their activity behaviour in response to wearing the device, there is the possibility that participants intentionally altered their activity behaviours (especially standing, LIPA, and MVPA). Finally, the majority of other research studies have included additional covariates such as nutritional status, socioeconomic status, stage of pubertal development, and parental health status. The use of additional covariates could add strength to the findings presented, although it may not be appropriate given the sample size presented.

## 6. Conclusion

The purpose of this research was to pilot methodologies for further empirical research. Although there is internationally accepted consensus on the importance of PA and exercise management for children with T1D, the results of this study show that this cohort of children with T1D is not achieving the recommended targets for living a physically active life with T1D. It is hoped that further empirical research will contribute to the development of additional education and intervention supports to aid the translation of ISPAD guidelines into real-world practice for PA promotion. Wearable PA trackers could play an important role in future research and PA promotion practices to aid in the transfer of PA guidelines to real-world changes in PA behaviours for this population. Larger-scale mixed-methods studies are required to advance our understanding of PA behaviours in children with T1D.

## Figures and Tables

**Table 1:** Summary of participant age (years), HbA1c level (mmol/mol), time since diagnosis (years), and insulin regimen (% pump, % injection) (mean ± std dev.).

Gender	*n*	Mean age (years) (±std dev.)	Mean weight (kg) (±std dev.)	Mean height (cm) (±std dev)	Mean HbA1c	Years since diagnosis (±std dev.)	Multiple daily insulin injection	Insulin pump therapy
Female	9	14.66 (±1.88)	72.26 (±24.28)	165.52 (±9.89)	67 mmol/Mol 8.25% (±1.13)	7.50 (±4.07)	75%	25%
Male	12	12.75 (±2.29)	66.53 (±9.08)	153.94 (±16.41)	64 mmol/Mol 7.97% (±1.91)	5.58 (±4.48)	62%	38%

**Table 2:** Comparison of daily steps across healthy weight and overweight BMI centile categories (mean ± std dev.).

Gender	Healthy weight (<85^th^ centile)	Mean daily steps (±std dev.)	Overweight/obese (>85^th^ centile)	Mean daily steps (±std dev.)	*p* value (effect size)
Female	57^th^ centile (*z* = −6.13) –85^th^ centile (*z* = 2.3)	7756 (±476)	86^th^ centile (*z* = −1.09) –99^th^ centile (*z* = 1.16)	4742 (±4065)	0.34 (0.67)
Male	31^st^ centile (*z* = −10.65) -83^rd^ centile (*z* = −1.61)	11742 (±5904)	88^th^ centile (*z* = −0.74) -95^th^ centile (*z* = 0.47)	10299 (±5544)	0.91 (0.80)

**Table 3:** Number of days during the 8-day wear protocol when the minimum recommended step count was achieved.

Gender	Mimimun recommended step count (11,500) achieved		
	0 days/8 days	1-3 days/8 days	4-8 days/8 days
Female	*n* = 1	*n* = 5	*n* = 3
Male	*n* = 0	*n* = 4	*n* = 8

**Table 4:** Comparison between male and female daily steps, standing time (minutes), and sitting time (minutes) (mean ± std dev.).

	Mean step count (±std dev.)	*p* value (effect size)	Mean standing time (m) (±std dev.)	*p* value (effect size)	Mean sitting time (m) (±std dev.)	*p* value (effect size)
Female	7306 (±5468)	*p* = 0.01 (0.77)	162 (±83.20)	*p* = 0.20 (0.25)	366 (±185.20)	*p* = 0.82 (0.05)
Male	10806 (±5578)		148 (±102)		335 (±135)	

**Table 5:** Comparison between accelerometer recorded weekend and weekday daily steps, standing time (minutes), and sitting time (minutes) (mean ± std dev.).

	Mean steps (±std dev.)	*p* value (effect size)	Mean standing time (m) (±std dev.)	*p* value (effect size)	Mean sitting time (m) (±std dev.)	*p* value (effect size)
Weekday	9126 (±6289)	*p* = 0.38 (0.03)	126 (±87.50)	*p* = 0.87 (0.39)	349 (±171.31)	*p* = 0.07 (0.19)
Weekend	7918 (±5130)		123 (±102)		418 (±198)	

**Table 6:** Comparison between male and female self-reported weekday and weekend MVPA duration (minutes).

Self-report weekday activity			Self-report weekend activity		
	Female	Male		Female	Male
None	0	0	None	0	0
1–30 mins	0	0	1–30 mins	0	0
31–60 mins	66%	53%	31–60 mins	98%	56%
1–2 hrs	34%	25%	1–2 hrs	0	0
2–3 hrs	0	0	2–3 hrs	2%	22%
3–4 hrs	0	0	3–4 hrs	0%	22%
>4 hrs	0	22%	>4 hrs	0	0

## Data Availability

The data that support the findings of this study are available from the corresponding author upon reasonable request.

## References

[1] Hawkes C. P., Murphy N. P. (2014). Paediatric type 1 diabetes in Ireland--results of the first national audit. *Irish Medical Journal*.

[2] The Task Force on diabetes, pre-diabetes, and cardiovascular diseases of the European Society of Cardiology (ESC) and developed in collaboration with the European Association for the Study of Diabetes (EASD) (2014). ESC guidelines on diabetes, pre-diabetes, and cardiovascular diseases developed in collaboration with the EASD - summary. *Diabetes and Vascular Disease Research*.

[3] World Health Organization (2019). *Global action plan on physical activity 2018-2030: more active people for a healthier world*.

[4] Rodriguez-Ayllon M., Cadenas-Sánchez C., Estévez-López F. (2019). Role of physical activity and sedentary behavior in the mental health of preschoolers, children and adolescents: a systematic review and meta-analysis. *Sports Medicine*.

[5] Chimen M., Kennedy A., Nirantharakumar K., Pang T. T., Andrews R., Narendran P. (2012). What are the health benefits of physical activity in type 1 diabetes mellitus? A literature review. *Diabetologia*.

[6] Quirk H., Blake H., Dee B., Glazebrook C. (2015). "having diabetes shouldn't stop them": healthcare professionals' perceptions of physical activity in children with type 1 diabetes. *BMC Pediatrics*.

[7] Valerio G., Spagnuolo M. I., Lombardi F., Spadaro R., Siano M., Franzese A. (2007). Physical activity and sports participation in children and adolescents with type 1 diabetes mellitus. *Nutrition, metabolism and cardiovascular diseases.*.

[8] Purnell J., Hokanson J. E., Marcovina S. M., Steffes M. W., Cleary P. A., Brunzell J. D. (1998). Effect of excessive weight gain with intensive therapy of type 1 diabetes on lipid levels and blood pressure: results from the DCCT. *JAMA*.

[9] Riddell M. C., Davis E. A., Mayer-Davis E. J., Kahkoska A., Zaharieva D. P. (2021). Advances in exercise and nutrition as therapy in diabetes. *Diabetes Technology & Therapeutics*.

[10] US Preventive Services Task Force (2017). Screening for obesity in children and adolescents: US Preventive Services Task Force Recommendation Statement. *JAMA*.

[11] Adolfsson P., Riddell M. C., Taplin C. E. (2018). ISPAD clinical practice consensus guidelines 2018: exercise in children and adolescents with diabetes. *Pediatric Diabetes*.

[12] DiMeglio L. A., Acerini C. L., Codner E. (2018). ISPAD Clinical Practice Consensus Guidelines 2018: Glycemic Control Targets and Glucose Monitoring for Children, Adolescents, and Young Adults with Diabetes. *Pediatric Diabetes*.

[13] Adams M. A., Johnson W. D., Tudor-Locke C. (2013). Steps/day translation of the moderate-to-vigorous physical activity guideline for children and adolescents. *International Journal of Behavioral Nutrition and Physical Activity*.

[14] Edwardson C. L., Winkler E. A. H., Bodicoat D. H. (2017). Considerations when using the activPAL monitor in field-based research with adult populations. *Journal of Sport and Health Science*.

[15] Dowd K. P., Harrington D. M., Bourke A. K., Nelson J., Donnelly A. E. (2012). The measurement of sedentary patterns and behaviors using the activPAL professional physical activity monitor. *Physiological Measurement*.

[16] Van der Berg J. D., Willems P. J., van der Velde J. H. (2016). Identifying waking time in 24-h accelerometry data in adults using an automated algorithm. *Journal of Sports Sciences*.

[17] Jose K. A., Blizzard L., Dwyer T., McKercher C., Venn A. J. (2011). Childhood and adolescent predictors of leisure time physical activity during the transition from adolescence to adulthood: a population based cohort study. *International Journal of Behavioral Nutrition and Physical Activity*.

[18] Li C., Chen X., Bi X. (2021). Wearable activity trackers for promoting physical activity: A systematic meta- analytic review. *International journal of medical informatics*.

[19] Pozzilli P., Guglielmi C., Caprio S., Buzzetti R. (2011). Obesity, autoimmunity, and double diabetes in youth. *Diabetes Care*.

[20] Liu L. L., Lawrence J. M., Davis C. (2010). Prevalence of overweight and obesity in youth with diabetes in USA: the SEARCH for diabetes in youth study. *Pediatric Diabetes*.

[21] Lipman T. H., Levitt Katz L. E., Ratcliffe S. J. (2013). Increasing incidence of type 1 diabetes in youth: twenty years of the Philadelphia pediatric diabetes registry. *Diabetes Care*.

[22] Nathan D. M., for the DCCT/EDIC Research Group (2014). The diabetes control and complications trial/epidemiology of diabetes interventions and complications study at 30 years: overview. *Diabetes Care*.

[23] Minges K. E., Whittemore R., Grey M. (2013). Overweight and obesity in youth with type 1 diabetes. *Annual Review of Nursing research*.

[24] Cuenca-Garcia M., Jago R., Shield J. P., Burren C. P. (2012). How does physical activity and fitness influence glycaemic control in young people with type 1 diabetes?. *Diabetic Medicine*.

[25] Stephenson A., McDonough S. M., Murphy M. H., Nugent C. D., Mair J. L. (2017). Using computer, mobile and wearable technology enhanced interventions to reduce sedentary behaviour: a systematic review and meta-analysis. *International Journal of Behavioral Nutrition and Physical Activity*.

